# Association of Altered Baseline Hematological Parameters with Adverse Tuberculosis Treatment Outcomes

**DOI:** 10.3390/pathogens14020146

**Published:** 2025-02-04

**Authors:** Arul Nancy Pandiarajan, Nathella Pavan Kumar, Kadar Moideen, Kannan Thiruvengadam, Syed Hissar, Shanmugam Sivakumar, Ramalingam Bethunaickan, Vijay Viswanathan, Hardy Kornfeld, Subash Babu

**Affiliations:** 1 National Institutes of Health-NIRT—International Center for Excellence in Research, Chennai 600 031, India; arul.p@icerindia.org (A.N.P.); kadarbinabbas@gmail.com (K.M.); sbabu@icerindia.org (S.B.); 2 ICMR-National Institute for Research in Tuberculosis, Chennai 600 031, India; tkannan1985@gmail.com (K.T.); syed.hissar@icmr.gov.in (S.H.); shanmugamsiva27@gmail.com (S.S.); bramalingam@gmail.com (R.B.); 3 Academy of Scientific and Innovative Research, Ghaziabad 201 002, India; 4 Prof. M. Viswanathan Diabetes Research Center, Chennai 600 013, India; drvijay@mvdiabetes.com; 5 University of Massachusetts Chan Medical School, Worcester, MA 01655, USA; hardy.kornfeld@umassmed.edu; 6 Laboratory of Parasitic Diseases (LPD), National Institute of Allergy and Infectious Diseases (NIAID), National Institutes of Health (NIH), Bethesda, MD 20892, USA

**Keywords:** tuberculosis, treatment outcomes, hematological parameters, ML ratio, NL ratio

## Abstract

Tuberculosis (TB) treatment monitoring is an essential tool for effective TB treatment management. Identifying parameters that predict adverse TB treatment outcomes could significantly improve clinical management. The association of hematological parameters with poor TB treatment outcomes is not well defined. To study the relationship of hematological parameters with TB treatment outcomes, we examined data from pulmonary tuberculosis (PTB) patients with successful (controls) and unsuccessful (cases) treatment outcomes. We enrolled 68 cases and 133 controls through a nested 1:2 case–control study, matching for age, sex, body mass index, diabetes status, alcohol and smoking. Hematological profiling showed significant difference in the absolute counts of white blood cells, lymphocytes, neutrophils and monocytes between cases and controls. In addition, increased neutrophil to lymphocyte ratio (NL) ratio and monocyte to lymphocyte (ML) ratio were present in cases in comparison to controls. Similarly, decreased hematocrit and red blood cell counts were detected in cases when compared with controls. Univariate and multivariate analysis demonstrated a significant association of absolute counts of WBC, neutrophils, monocytes, NL and ML ratios with poor treatment outcomes. The altered baseline hematological parameters are clearly associated with the poor TB treatment outcomes, showing potential for clinical prediction to enhance management of at-risk cases.

## 1. Introduction

Tuberculosis (TB) is a devastating infectious disease with the highest mortality rate, claiming 1.6 million lives worldwide with a 4.5% increase globally [1]. Despite the global execution of the DOTS (directly observed therapy short-course) program, TB has remained a high-burden disease for the past three decades [2]. India is one of the eight countries with the highest burden of new TB cases [1], and the current incidence rate in India had a 19% increase in 2021 [3]. The high prevalence of TB is due to various risk factors like age, gender, HIV, diabetes, household crowding, indoor air pollution, immune status and lifestyle habits like smoking, alcoholism and work-related risks [4]. Published studies have reported that 57% of TB patients were underweight, 88% were anemic, and 48% of the anemia cases were due to iron deficiency [5]. However, our research revealed no significant differences in hematological parameters among latent TB individuals, irrespective of their nutritional status [6,7]. Unfavorable or adverse treatment outcomes in TB include recurrence, failure and loss of follow-up. Recurrent TB includes both relapse and re-infection, which are second episodes of TB disease where identical isolates lead to relapse and non-identical isolates lead to re-infection. Smear-positive relapse cases are around 24% among all the smear-positive cases that hamper TB treatment management [8]. A successful TB treatment is the cure of TB disease, which acts as an indicator for the effective management of the End TB strategy [1]. 

Currently available bacteriological examinations like sputum smear and culture grade are still the confirmatory tests to determine the presence of active pulmonary tuberculosis (PTB) and to monitor the degree of disease progression [9]. They are time-consuming with poor sensitivity. Radiological investigations like chest X-ray (CXR) highlight the severity of the disease through cavitation or other pathology in the lungs [10]. Other molecular tests like GeneXpert and LAMP assay are more sensitive than sputum smear culture but quite expensive and exhibit the cumbersome process of primer design [11,12]. The existing baseline diagnostic tools have limitations, as they are not efficient to determine the early prediction of treatment outcomes. 

Hematological perturbation is common in PTB and has been evaluated as a diagnostic and prognostic marker [13]. Very few studies have been conducted on the impact of the hematological profile that determine adverse TB treatment outcomes. Hence, we wanted to elucidate whether hematological parameters could serve as predictive biomarkers for poor or adverse PTB treatment outcomes in this population.

## 2. Materials and Methods

### 2.1. Study Population

The subjects in this study were recruited upon approval obtained from the Ethics Committees of the Prof. M. Viswanathan Diabetes Research Center and National Institute for Research in Tuberculosis (NIRT) with proper informed consent. The subjects were from Chennai, South India, and enrolled in the prospective Effect of Diabetes on Tuberculosis Severity (EDOT) study. Adults in the age group of 20 to 75 with new sputum smear and culture positives, as well as positive by NAAT, were included in the study. Positive sputum culture on solid medium (Lowenstein–Jensen media) with an appropriate chest X-ray confirmed the diagnosis of PTB. In addition, X-ray scores were also recorded for all the participants. Individuals with any previous episodes of TB disease, TB treatment in the past, drug-resistant TB, positive HIV status, under immune suppression medications and pregnant and lactating women were excluded from the study. All the recruited pulmonary TB patients were given anti-TB treatment in accordance with End TB strategy, as recommended by the WHO, which was successfully managed by the NTEP (National Tuberculosis Elimination Program) [14]. The subjects were followed up throughout and after treatment, starting from month 6 and continuing until 1 year after the completion of the treatment. The current hematological study is retrospective. Treatment outcome was classified into adverse (poor) and good treatment outcomes, in which good refers to microbiological cure and “adverse” refers to treatment failure, recurrence and death. The microbiological cure was diagnosed by negative sputum culture at months 5 and 6 of TB treatment, whereas treatment failure was diagnosed as positive sputum culture at month 5 or month 6. Individuals with good treatment outcomes were named as controls, and those with adverse treatment outcomes as cases. Here, we have 68 cases and 133 controls, which were obtained through a nested case–control matching of cases to controls in a 1:2 ratio for age, sex, body mass index (BMI) and diabetes status. The demographic and epidemiological data have been previously reported [15].

### 2.2. Hematological Parameters

Around 2 mL of blood was collected intravenously in EDTA (ethylene diamine tetra acetic acid)-coated tubes and was analyzed in Beckman Coulter DxH520, an automated hematology analyzer. The complete blood count (CBC) includes the relative and absolute count of RBC (red blood cell), WBC (white blood cells), lymphocytes, eosinophil, neutrophils, monocytes, basophils, platelets, hematocrit and Hb estimation. Apart from this, NLR and MLR were calculated with the obtained cell counts.

### 2.3. Statistical Data Analysis

Statistical analysis was performed using GraphPad PRISM V.9.0. Statistical significance was considered if the *p*-value was < 0.05. To compare the groups, the Mann–Whitney test was applied. The median and interquartile range were used as the measure of central tendency and dispersion. A univariate model was developed to study the association of hematological parameters with poor TB treatment outcomes. Multivariate logistic regression analysis was carried out by adjusting multiple variables, including age, gender, body mass index, diabetic status, alcoholism, smoking and smear grade status. Both models were performed using IBM SPSS Statistics 25 (2017, IBM Corporation, Armonk, NY, USA) Spearman’s correlation was performed using the R studio (2021.09.2+382) platform. The combination of predictive biomarkers was determined by using an online application service (CombiROC v1.2).

## 3. Results

### 3.1. Characteristics of the Study Population

The demographics of the study population are shown in Table 1. There were no significant differences in terms of age, gender, diabetes status, smear and culture grade, BMI and lifestyle risk factors like smoking and alcoholism between these two groups of TB treatment outcomes (Table 1).

### 3.2. Association of Hematological Parameters with Poor Treatment Outcomes

**Figure 1:**
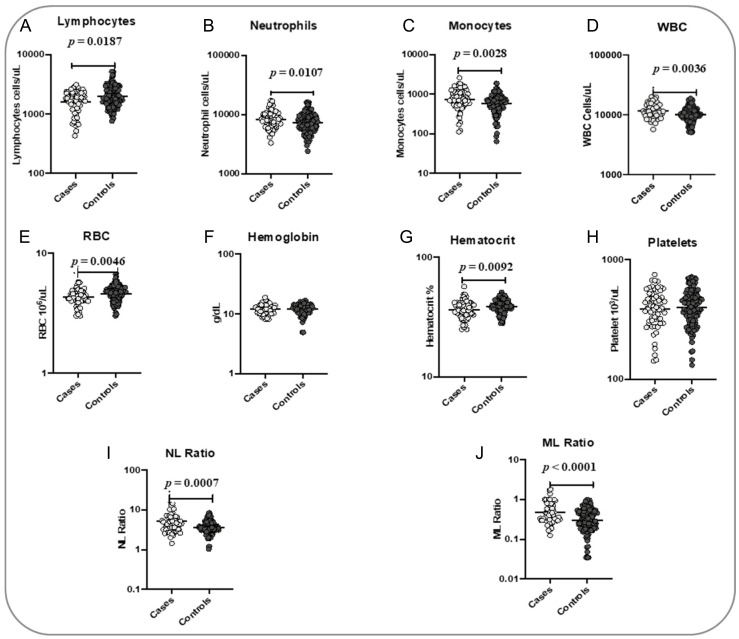
Association of hematological parameters with poor treatment outcomes. The baseline hematological parameters were measured in cases (n = 68) and controls (n = 133). The data are represented as scatter plots, with each circle representing a single individual. *p* values were calculated using the Mann–Whitney test with Holm’s correction for multiple comparisons. The hematological parameters measured are as follows: (**A**) Lymphocytes; (**B**) Neutrophils; (**C**) Monocytes; (**D**) WBC; (**E**) RBC; (**F**) Hemoglobin; (**G**) Hematocrit; (**H**) Platelets; (**I**) NL Ratio; (**J**) ML Ratio.

To determine the association of hematological parameters with the TB treatment outcome, we measured the baseline hematological parameters, including Hb, RBC, hematocrit, platelets, absolute WBC, neutrophil, lymphocyte and monocyte counts (Figure 1). The RBC count (*p* = 0.0046), hematocrit (*p* = 0.0092) and absolute lymphocyte count (*p* = 0.0187) were significantly lower in cases when compared with the controls. Furthermore, the absolute WBC (*p* = 0.0036), neutrophil (*p* = 0.0107) and monocyte counts (*p* = 0.0028) were significantly higher in cases than in controls. The NLR (*p* = 0.0007) and MLR (*p* = 0.0001) were also significantly higher in cases compared to controls. Hb and platelet levels did not show any significant difference between the groups (Figure 1).

### 3.3. Logistic Regression Analysis of the Association of Hematological Parameters with Treatment Outcomes

Univariate and multivariate analysis revealed that some of the hematological parameters are strongly associated with poor treatment outcomes without the influence of the other confounding factors. Interestingly, in the univariate analysis, we could see the significance in the absolute counts of WBC (OR, 3.51; 95% CI, 1.62–7.59; *p* = 0.001), neutrophils (OR, 2.19; 95% CI, 1.20–3.98; *p* = 0.011), monocytes (OR, 1.68; 95% CI, 1.14–2.47; *p* = 0.008), NLR (OR, 2.68; 95% CI, 1.68–4.26; *p* < 0.001) and MLR (OR, 2.32; 95% CI, 1.59–3.39; *p*< 0.001), and they were all strongly associated with a higher risk of adverse treatment outcomes. In contrast, RBC (OR, 0.14; 95% CI, 0.03–0.55; *p* = 0.005), hematocrit (OR, 0.13; 95% CI, 0.03–0.55; *p* = 0.006) and absolute lymphocyte count (OR, 0.44; 95% CI, 0.27–0.74; *p* = 0.002) were associated with a lower risk of adverse treatment outcomes. Multivariate analysis further confirmed the above significance with the adjusted odd ratio of the absolute counts of WBC (aOR, 3.14; 95% CI, 1.39–7.08; *p*= 0.006), neutrophils (aOR, 1.91; 95% CI, 1.01–3.62; *p*= 0.046), monocytes (aOR, 1.63; 95% CI, 1.08–2.46; *p*= 0.019), NLR (aOR, 2.52; 95% CI, 1.55–4.09; *p* < 0.001) and MLR (aOR, 2.30; 95% CI, 1.54–3.45; *p* < 0.001), all associated with a high risk of adverse treatment outcomes. In multivariate analysis, RBC (aOR, 0.15; 95% CI, 0.04–0.65; *p* = 0.011), hematocrit (aOR, 0.14; 95% CI, 0.03–0.65; *p* = 0.012) and absolute lymphocyte count (aOR, 0.45; 95% CI, 0.26–0.77; *p*= 0.003) were associated with a lower risk of adverse treatment outcomes (Table 2).

### 3.4. Baseline Signature of Two or Three Hematological Parameters Could Be a Predictive Biomarker Discriminating Adverse TB Treatment Outcome from PTB Cured Controls

**Figure 2:**
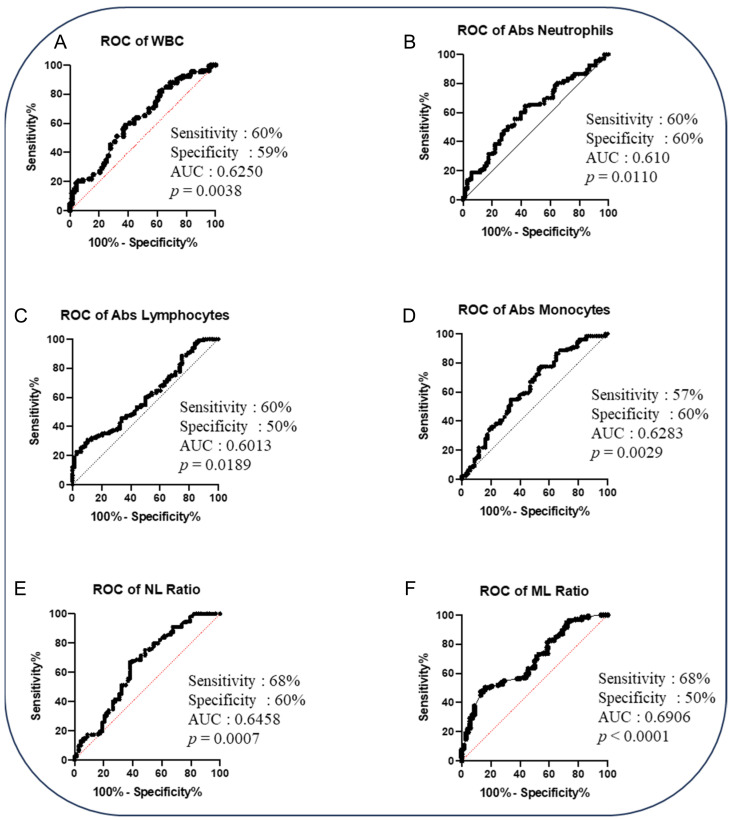
The baseline hematological parameters could predict adverse TB treatment outcomes in PTB patients. Altered hematological parameters with cases (n = 68) from cured controls (n = 133). Receiver operator characteristic (ROC) analysis is to evaluate the sensitivity, specificity and area under the curve (AUC) to estimate the capability of the hematological parameters to distinguish favorable versus unfavorable treatment outcomes. The receiver operator characteristics of hematological parameters are as follows: (**A**) ROC of WBC; (**B**) ROC of absolute neutrophils; (**C**) ROC of absolute lymphocytes; (**D**) ROC of absolute monocytes; (**E**) ROC of NL ratio; (**F**) ROC of ML ratio.

**Figure 3:**
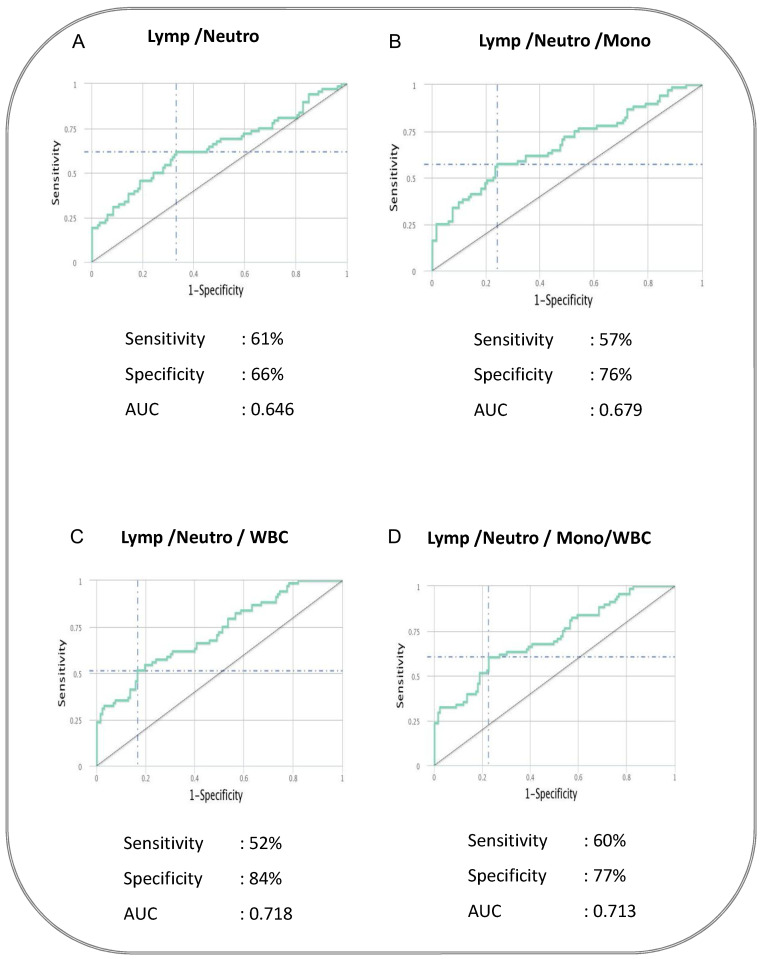
The baseline combination of hematological parameters could be a predictive biomarker for adverse TB treatment outcomes in PTB patients. Combination of hematological parameters showing its association with adverse treatment outcomes in active pulmonary tuberculosis patients. The combination of receiver operator characteristic (Combi-ROC) analysis moderately discriminates cases from controls. The dual combination is shown in (**A**) Lymphocytes/Neutrophils; the triple combination is shown in (**B**) Lymphocytes/Neutrophils/Monocytes and (**C**) Lymphocytes/Neutrophils/WBC; the quadruple combination was shown in (**D**) Lymphocytes/Neutrophils/Monocytes/WBC.

To determine whether we could deduce a hematological signature that could be used as a predictive biomarker for cases versus controls, we performed an ROC analysis and a combined ROC analysis. The ROC curve of logistic regression model exhibited AUC of 0.646, sensitivity of 68% and specificity of 60% in NL Ratio, whereas in ML Ratio, it presented AUC of 0.691, sensitivity of 68% and specificity of 50% to distinguish cases from controls (shown in Figure 2). ROC analysis of various combinations of hematological parameters between the absolute count of WBC, lymphocytes, neutrophils and monocytes displayed a good AUC (0.646–0.718) with sensitivity (52–61%) and specificity (66–84%) to distinguish cases from cured controls (shown in Figure 3). Thus, alteration in the hematological parameters can be further evaluated as a biomarker to predict adverse treatment outcomes.

### 3.5. Baseline Hematological Parameters Are Weakly Correlated with the Chest X-Ray Score in Active PTB Patients

**Figure 4:**
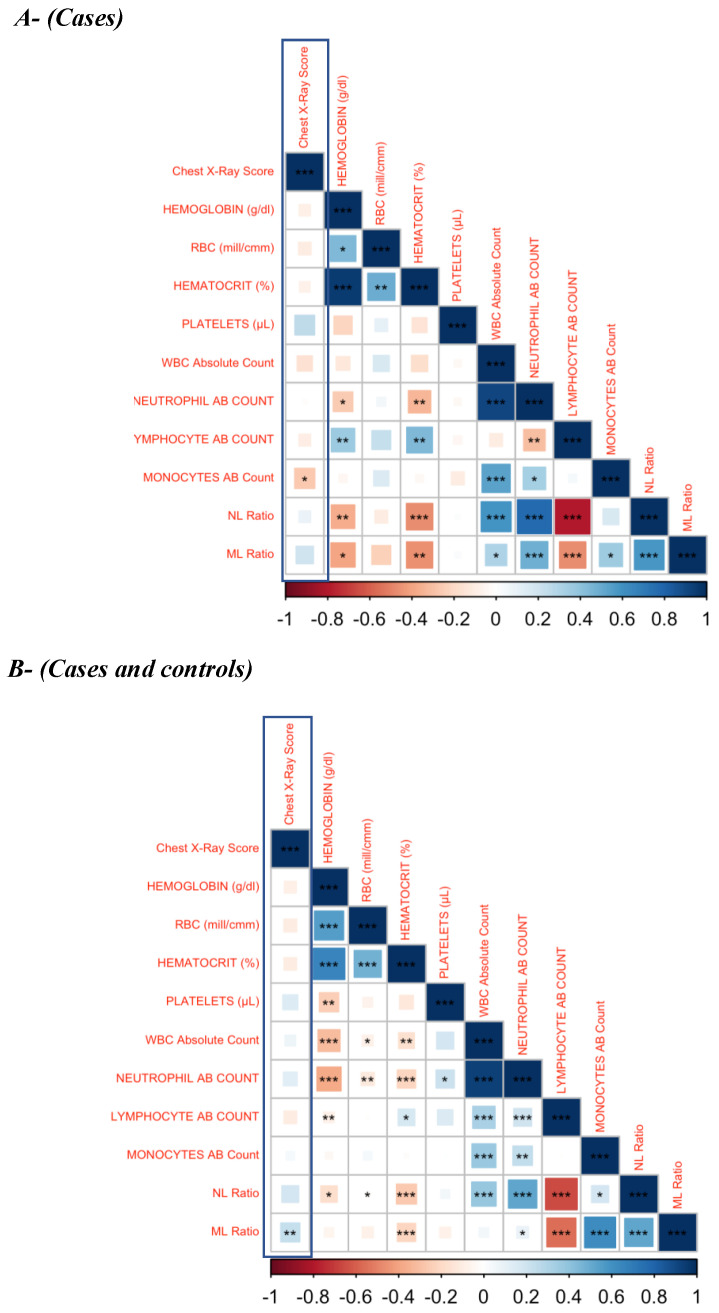
Correlation Matrix. (**A**)—cases; (**B**)–cases and cured controls. The baseline hematological parameters are weakly associated with the chest X-ray score in active PTB patients. Weak association of baseline hematological parameters (Hb, hematocrit, platelet, RBC, absolute count of WBC, neutrophil, lymphocyte, monocyte and NL ratio) with the chest X-ray score was found in active PTB patients. There was a significant but weak association between absolute monocyte count in cases and ML ratio in both cases and cured controls with the chest X-ray score. *p* value—* <0.05; ** <0.01; *** <0.001.

Since lung pathology plays a crucial role in all of the adverse treatment outcomes, we examined the association of hematological parameters with the chest X-ray score shown in Figure 4 (A—cases and B—combined cases and controls). The monocyte absolute count was statistically significant and weakly correlated in cases, with a *p*-value of 0.038 (Figure 4A). 

The ML (*p* = 0.0016) ratio was also weakly associated with chest X-ray score in the combined status of cases and cured control. A negative and weak association of CXR score with other parameters, such hemoglobin, hematocrit, RBC, absolute count of WBC, neutrophil, lymphocyte and monocytes, was observed in cases, with no statistical significance (Table 3 and Table 4).

## 4. Discussion

The ability to identify those individuals at risk for adverse outcomes at the time of diagnosis could be used to target novel strategies to enhance results and improve TB treatment outcomes. There are many underlying risk factors for adverse treatment outcomes, including the presence of drug-resistant bacteria, lifestyle habits like smoking and alcoholism, co-morbidities like diabetes and HIV and low BMI [16]. Treatment failure is known to be higher in PTB than in extrapulmonary TB [17]. Hence, it is essential to elucidate the host immune response and virulence of the microorganism that leads to adverse treatment outcomes. Hematological profiling is widely available and performed in diverse clinical studies. Several studies reported that decreased RBC, WBC and platelet counts, as well as serum Hb, increased erythrocyte sedimentation rate (ESR) and C-reactive protein are commonly observed in TB patients [18]. Hematological parameters were identified as a biomarker in the diagnosis and follow-up of PTB patients co-infected with HIV [19]. Low Hb levels also act as an indicator of TB disease severity before TB treatment [20]. Evidence has accumulated in recent years that blood cell counts, in combination with clinical manifestations, can predict TB disease progression and treatment outcomes [13,21]. Most of the previous studies focused on neutrophil-to-lymphocyte (NLR) ratio, monocyte-to-lymphocyte (MLR) ratio, erythrocyte sedimentation rate (ESR) and Hb level. All these hematological parameters help determine disease severity [22], associated with recurrence, co-morbid conditions and mortality rate [23]. Our study on the association of hematological parameters with TB treatment outcomes reveals similar results, with diminished RBC count and elevated WBC count in cases compared to cured controls.

Most of the previous studies reported that PTB patients exhibit diminished levels of Hb, WBC count [24], packed cell volume (PCV), mean corpuscular Hb and mean corpuscular volume [25], as well as CD4 count [26], elevated ESR, mean cell Hb concentration, relative plasma viscosity and euglobulin lysis time [27]. Moreover, certain hematological alterations like CD4 lymphopenia, neutropenia, thrombocytopenia, anemia and thrombocytosis were predominant only in PTB patients and not in PTB with HIV [19,28]. This shows the unique signature of hematological parameters in PTB patients that are not influenced by HIV. In the present study, we found no significant difference in Hb and platelets between cases and controls.

Pre-treatment NLR and MLR were recommended as clinical diagnostic tools related to TB disease severity [29]. Upon TB treatment, MLR and NLR were significantly diminished and correlated with culture-negative status [30]. Our present study complements these data, as MLR and NLR were higher at baseline in cases than in controls. In a randomized clinical study of the South Indian population, it was reported that hematological parameters like percentages of Hb, neutrophils and lymphocytes greatly change in newly diagnosed PTB patients after anti-TB treatment, which reveals the impact of ATT in improving the immune response [31]. Newly diagnosed PTB patients displayed altered hematological parameters, such as elevated ESR and platelet count, diminished packed cell volume and Hb [32]. Our current study does not show any statistical significance in platelet and Hb between the cases and the cured controls.

Re-treatment of drug sensitive PTB was predicted by the initial radiological presentation [33]. In this study, there was a significant but weak association between the baseline hematological parameters and chest X-ray in cases (absolute count of monocyte) and the combined status of both cases and cured controls (ML ratio). Other hematological parameters with no statistical significance and a weak association with the CXR score showed that the baseline hematological profile predicts unfavorable TB treatment outcomes even without the influence of the CXR score. However, incorporating the CXR score is likely to enhance the predictive value of the model that includes hematological parameters.

Most of our results are concordant with previous studies, highlighting the utility of analyzing the association of hematological parameters between good and poor TB treatment outcomes in clearly defined and clinically relevant study groups in the Indian population. The results of our study and others cited here provide a basis for designing diagnostic and, ultimately, interventional trials seeking to leverage baseline predictors of unfavorable TB treatment outcomes to stratify at-risk individuals for enhanced management strategies. Despite the value of the results, our study suffers from certain limitations, as it only includes baseline hematological data. Although the sample size seems to be moderate, these results were not validated with an external cohort from a different ethnic population. The ultimate goal is to safeguard PTB patients at risk of failing TB treatment or experiencing recurrence by utilizing a routine blood cell count. When integrated with clinical algorithms, this approach could serve as a simple yet effective tool for treatment monitoring. By combining complete blood count data with existing microbiological and radiological markers, along with emerging cytokine profiles, healthcare providers could better stratify PTB patients at risk of treatment failure or recurrence. This proactive strategy would enable the identification of vulnerable populations and facilitate the provision of additional clinical care well in advance.

## 5. Conclusions

In summary, baseline hematological parameters show alterations that are associated with TB treatment outcomes, warranting further validation.

## Figures and Tables

**Table 1:** Demographic and clinical characteristics of the study population.

Parameters	Cases (n = 133)	Controls (n = 68)	*p*-Value
Age in years	45 (36–50)	45 (38–52)	0.268
Gender			
Female	23 (17.3)	8 (11.8)	0.305
Male	110 (82.7)	60 (88.2)	
BMI	18 (1620)	17 (15–19)	0.149
Diabetes Status			
Non-Diabetes	59 (44.4)	26 (38.2)	0.406
Diabetes	74 (55.6)	42 (61.8)	
Cough			
Absence	2 (1.5)	1 (1.5)	0.985
Presence	131 (98.5)	67 (98.5)	
Dyslipidaemia			
Absence	133 (100)	68 (100)	NA
Presence	0 (0)	0 (0)	
Smoking			
Never	74 (55.6)	26 (38.2)	0.034
Past	26 (19.5)	14 (20.6)	
Current	33 (24.8)	28 (41.2)	
Alcohol			
Never	42 (31.6)	16 (23.5)	0.466
Past	25 (18.8)	13 (19.1)	
Current	66 (49.6)	39 (57.4)	
Cavity			
Absence	78 (58.6)	40 (58.8)	>0.990
Presence	36 (27.1)	18 (26.5)	
Not Known	19 (14.3)	10 (14.7)	
Smear ^a^			
1+	90 (67.7)	36 (52.9)	0.057
2+	40 (30.1)	27 (39.7)	
3+	3 (2.3)	5 (7.4)	
Culture ^b^			
1+	61 (45.9)	25 (36.8)	0.158
2+	26 (19.5)	10 (14.7)	
3+	46 (34.6)	33 (48.5)	
Chest X-ray Score median (IQR)	38 (5–130)	37 (2–125)	0.1943

The values were represented as n (%) and median (first–third quartile); Fisher Exact and Mann–Whitney test were used to check the significance. Abbreviations: BMI—body mass index (calculated as weight in kilograms divided by height in meters squared); DM—diabetes mellitus. a—Under 200× magnification, 1+ indicates 3 to 24 acid-fast bacilli (AFB) in 1 field, 2+ indicates 25 to 250 AFB in 1 field, 3+ indicates more than 250 AFB in 1 field. b—Under 200× magnification, 1+ indicates 10 to 100 colonies, 2+ indicates more than 100 to 200 colonies and 3+ indicates more than 200 colonies.

**Table 2:** Univariate and multivariate analysis for hematological profile.

Marker	Univariable Model		Multivariable Model	
	OR (95% CL)	*p*-Value	aOR * (95% CL)	*p*-Value
Hemoglobin (g/dl)	0.91 (0.34–2.41)	0.85	0.91 (0.31–2.63)	0.859
RBC (mill/cm)	0.14 (0.03–0.55)	**0.005**	0.15 (0.04–0.65)	0.011
Haematocrit	0.13 (0.03–0.55)	**0.006**	0.14 (0.03–0.65)	0.012
Platelets (ul)	0.84 (0.47–1.49)	0.541	0.85 (0.46–1.56)	0.593
WBC absolute count	3.51 (1.62–7.59)	**0.001**	3.14 (1.39–7.08)	**0.006**
Neutrophil absolute count	2.19 (1.20–3.98)	0.011	1.91 (1.01–3.62)	0.046
Lymphocyte absolute count	0.44 (0.27–0.74)	**0.002**	0.45 (0.26–0.77)	**0.003**
Monocyte absolute count	1.68 (1.14–2.47)	0.008	1.63 (1.08–2.46)	0.019
NL Ratio	2.68 (1.68–4.26)	**<0.001**	2.52 (1.55–4.09)	**<0.001**
ML Ratio	2.32 (1.59–3.39)	**<0.001**	2.30 (1.54–3.45)	**<0.001**

CI—confidence interval; OR—odds Ratio; aOR—adjusted odds ratio; * Multivariate logistic regression models study the association of biomarker with treatment outcomes (unfavorable), and are adjusted for age in years, gender, body mass index, diabetes status, smoking status, alcohol status and smear grading. Bold numbers mean statistically significant values.

**Table 3:** Relationship of hematological parameter with chest X-ray score in cases.

Chest X-Ray Score	*p*-Value	R Value	Correlation
HEMOGLOBIN (g/dl)	0.654	−0.083	Very Weak
HEMATOCRIT (%)	0.741	−0.073	Very Weak
PLATELETS (µL)	0.064	0.259	Weak
RBC	0.239	−0.103	Weak
WBC Absolute Count	0.29	−0.151	Weak
NEUTROPHIL Absolute Count	0.616	−0.01	Very Weak
LYMPHOCYTE Absolute Count	0.285	−0.091	Weak
MONOCYTES Absolute Count	0.039 *	−0.269	Weak
NL Ratio	0.336	0.089	Very Weak
ML Ratio	0.061	0.2	Weak

*p*-value—* <0.05.

**Table 4:** Relationship of hematological parameter with chest X-ray score in cases and cured controls.

Chest X-Ray Score	*p*-Value	R Value	Correlation
HEMOGLOBIN (g/dl)	0.144	−0.09	Very Weak
HEMATOCRIT (%)	0.088	−0.101	Very Weak
PLATELETS (µL)	0.067	0.146	Very Weak
RBC	0.058	−0.106	Very Weak
WBC Absolute Count	0.421	0.074	Very Weak
NEUTROPHIL Absolute Count	0.072	0.123	Very Weak
LYMPHOCYTE Absolute Count	0.389	−0.104	Very Weak
MONOCYTES Absolute Count	0.978	0.045	Very Weak
NL Ratio	0.055	0.183	Very Weak
ML Ratio	0.002 **	0.236	Weak

*p*-value—** <0.001.

## Data Availability

All data generated or analyzed during this study are included in this published article.

## Abbreviations

TB	tuberculosis
PTB	pulmonary tuberculosis
WBC	white blood cells
ML	monocyte to lymphocyte
NL	neutrophil to lymphocyte
DOTS	Directly observed therapy short-course
CXR	Chest X-ray
NIRT	National Institute for Research in Tuberculosis
EDOT	Effect of Diabetes on Tuberculosis Severity
NTEP	National Tuberculosis Elimination Program
BMI	body mass index
CBC	Complete blood count
aOR	adjusted odds ratio
Hb	Hemoglobin
ESR	erythrocyte sedimentation rate
PCV	packed cell volume
